# Production of Rigid Polyurethane Foams Using Polyol from Liquefied Oil Palm Biomass: Variation of Isocyanate Indexes

**DOI:** 10.3390/polym13183072

**Published:** 2021-09-11

**Authors:** Umar Adli Amran, Kushairi Mohd Salleh, Sarani Zakaria, Rasidi Roslan, Chin Hua Chia, Sharifah Nabihah Syed Jaafar, Mohd Shaiful Sajab, Marhaini Mostapha

**Affiliations:** 1 Bioresources and Biorefinery Laboratory, Materials Science Program, Faculty of Science and Technology, Universiti Kebangsaan Malaysia, Bangi 43600, Selangor, Malaysia; umaradliamran90@gmail.com (U.A.A.); chia@ukm.edu.my (C.H.C.); nabihah@ukm.edu.my (S.N.S.J.); 2 Faculty of Industrial Sciences and Technology, Universiti Malaysia Pahang, Lebuhraya Tun Razak, Gambang 26300, Pahang, Malaysia; rasidi@ump.edu.my; 3 Research Centre for Sustainable Process Technology (CESPRO), Department of Chemical and Process Engineering, Faculty of Engineering and Built Environment, Universiti Kebangsaan Malaysia, Bangi 43600, Selangor, Malaysia; mohdshaiful@ukm.edu.my; 4 Higher Institution Centres of Excellence, Center for Biofuels and Biochemical Research, Institute of Self-Sustainable Building, Universiti Teknologi PETRONAS, Bandar Seri Iskandar 32610, Perak, Malaysia; marhaini.mostapha@utp.edu.my

**Keywords:** polyol, empty fruit bunch, liquefaction, cellular polymer, compressive

## Abstract

Development of polyurethane foam (PUF) containing bio-based components is a complex process that requires extensive studies. This work reports on the production of rigid PUFs from polyol obtained via liquefaction of oil palm empty fruit bunch (EFB) biomass with different isocyanate (NCO) indexes. The effect of the NCO index on the physical, chemical and compressive properties of the liquefied EFB-based PUF (EFBPUF) was evaluated. The EFBPUFs showed a unique set of properties at each NCO index. Foaming properties had affected the apparent density and cellular morphology of the EFBPUFs. Increasing NCO index had increased the crosslink density and dimensional stability of the EFBPUFs via formation of isocyanurates, which had also increased their thermal stability. Combination of both foaming properties and crosslink density of the EFBPUFs had influenced their respective compressive properties. The EFBPUF produced at the NCO index of 120 showed the optimum compressive strength and released the least toxic hydrogen cyanide (HCN) gas under thermal degradation. The normalized compressive strength of the EFBPUF at the NCO index of 120 is also comparable with the strength of the PUF produced using petrochemical polyol.

## 1. Introduction

Polyurethane (PU) is a versatile polymer and widely used in various products. In general, PU is synthesized using polyols and diisocyanates. Polyols are organic chemical compounds comprising multiple hydroxyl (OH) groups. Polymerization of polyol and diisocyanate occurs when the OH groups react with isocyanate (NCO) groups, producing a polymer with monomer units linked by urethane linkages, called PUs. Formulations can be tailored to produce thermoplastic or thermosetting PUs for different applications, including, for example, adhesives, elastomers, coatings, sealants, films, and foams [1]. Polyurethane consumption in both the United States of America and Europe was estimated at about 6–8% of the total plastics usage for the region in 2019, and PU foams (PUFs) represented the largest segment of the total PU consumption [2,3]. Mainly, PUFs are categorized into flexible, semi-rigid, and rigid PUFs. Rigid PUF constitutes ca. 50% of the total worldwide production of PUFs [4].

Most polyols and PUs in the current market are made using petrochemicals. Increasing awareness on the depletion of non-renewable resources has triggered efforts for development of polyols and PUs from renewable and sustainable materials. Presently, lignocellulose biomass, protein, and plant-derived oils are utilized to partially substitute or/and replace petro-chemical-based polyols [5,6,7]. Lignocellulosic materials, such as industrial crops biomass and timber wastes, are abundant, cheap, and sustainable resources. Major components of lignocellulose, such as cellulose, hemicellulose, and lignin, store abundant natural OH compounds. Hence, lignocellulose has become a compelling alternative and substitute for petrochemicals in manufacturing of polyols.

Palm oil has been used in the production of diverse products, i.e., foods, pharmaceuticals, soaps, biofuels, and lubricants. The total global supply of palm oil in 2019 was estimated at 72,271,000 metric tons, where Indonesia and Malaysia were two major suppliers, sharing the market by 58.8% and 25.6%, respectively [8]. Production of palm oil releases a variety of byproducts and biomass wastes that possess economic values. Palm oil is extracted via pressing of oil palm fresh fruit bunch (FFB). The remnant of pressed FFB, namely empty fruit bunch (EFB), is one of the main lignocellulosic biomasses produced in palm oil refineries. Malaysia harvested 101.4 million tons of FFB from 5.9 million hectares of oil palm plantation countrywide in 2019 [9]. Since the wet weight of EFB is estimated to be ≈23.0% of the weight of FFB [10], relatively more than 23.3 million tons of EFB was produced by the industry for that year. To date, EFB has been used as a mulch for plantation, an additive in ruminant palletized foods, a filler for composites, and as fuel for generation of electricity. However, this biomass is still underutilized and it has other unexploited potentials.

Lignocellulosic biomass can be converted into liquefied bio-based chemicals via thermochemical process, namely liquefaction [1]. Alcohols and phenol are frequently used as liquefying solvents for preparation of polyols [11,12] and phenolic derivatives [13,14]. Liquefied biomass polyols and phenolic derivatives have been used in the production of PU [15] and phenolic [16,17] polymers, respectively. Liquefaction in acidified polyhydric alcohol causes depolymerization of biomass macromolecules into micromolecules via catalytic solvolysis reaction. Previously, bio-based polyol has been produced through direct liquefaction of EFB fibers using polyhydric alcohol as a liquefying solvent [18,19].

Lignocellulosic biomass contains high moisture levels, which could affect the properties of the converted bio-based chemicals or fuels. Processes to completely eradicate the moisture content often require high energy and are not economically feasible. Moisture trapped in the polyol produced from liquefied lignocellulosic biomass would react with NCO in the synthesis of PU, intervening in the polymerization of the polyol and NCO. Reaction of water and NCO produces amines and carbon dioxide (CO_2_) and emits heat. The polymerization and the release of CO_2_, which coincide during the polyol-isocyanate reaction, would produce PU with porosity. Therefore, lignocellulosic biomass-based polyol is more suitable to be used for production of PUFs.

Several researchers have produced rigid PUFs using polyols derived from the liquefaction of various lignocellulose biomass, such as cork [20], wheat straw [21], spent coffee ground [22], and peanut shells [23]. Although the properties of the liquefied lignocellulose polyols are comparable, the utilization of different types of diisocyanate and other formulations in the making of the PUFs have resulted in a wide range of compressive strengths and moduli. In general, the compressive strength of the PUFs increases with their densities. However, the amount of opened- and closed-cells of the PUFs also affects the compressive strength, causing denser PUFs to exhibit lower compressive strength than the less dense PUFs, and vice versa. Abdel Hakim et al. (2011) found that PUFs prepared from a binary mixture of liquefied lignocellulose (30% liquefied sugarcane biopolyol) and petro-chemical polyol (70% PEG) had shown higher compressive strength and thermal stability than that of petrochemical-based PUFs [24]. This is due to the higher crosslinking density in the PUFs produced from the mixed polyols system, contributed by the combination of high OH content of the petro-chemical polyol and enhanced crosslinking by lignin component products in the liquefied lignocellulose polyol. Meanwhile, PUFs that were produced solely using liquefied lignocellulose-based polyol showed unique sets of properties that were influenced by each property of polyol, such as OH content, molecular weight, and functionality [1]. Normally, a polyol produced from liquefaction of lignocellulose biomass contains lower OH content than that of petro-chemical polyols. Thus, PUFs produced from 100% biomass-based polyol would have lower crosslinking density and compressive strength than the PUFs produced from mixture of petro-chemical and liquefied lignocellulose polyols [1]. These circumstances require studies to be carried out for utilization of different biomass-based polyols.

Stoichiometric studies on different molar ratio of NCO/OH are important in determining the properties of PUFs. NCO index (NCO equivalent / OH equivalent × 100) is widely used to represent the molar ratio of NCO/OH in production of PUFs. Different NCO indexes can affect the fraction of hard segments (urethane and/or urea linkages) and the formation of inter- and intra-hydrogen bonding in the PU network, which could influence its strength and thermal stability [25]. Kim et al. (2008) found that different NCO indexes affected the foaming properties of PUFs [26]. Hejna et al. (2017) reported that increases in the NCO index had affected the crosslink density of PUF [27]. Mishra et al. (2012) discovered that the fraction of hard segments (urethane and/or urea linkages) and formation of inter- and intra-hydrogen bonding in the PU network had been affected by varying NCO indexes [25]. They also observed that the hydrophobicity of the polymer increased with the NCO index. In addition, variation of the NCO index can greatly affect the phase separation of hard and soft segments in the PU network [28]. In this study, liquefied EFB polyol was used to prepare rigid PUFs with different NCO indexes to evaluate the effect of the NCO index on the physical and chemical properties of the EFB polyol-based PUFs (EFBPUFs).

## 2. Materials and Methods

### 2.1. Materials

Oil palm EFB fiber was supplied by the Malaysian Palm Oil Board (MPOB). The EFB fiber was ground and sieved through a 250–300 micron screen, then dried at 105 °C for 24 h before use. Polyethylene glycol (PEG) with an average molecular weight (M_w_) of ≈400, glycerol (Gly), sulfuric acid (95–98% purity H_2_SO_4_), and magnesium oxide (MgO) were purchased from R&M Chemicals. 1,4-dioxane (99.0%), sodium hydroxide (96.0% purity NaOH), 1,4-diazabicyclo[2,2,2]octane (DABCO), pyridine (99% purity), phthalic anhydride, and imidazole (99.0%) were acquired from Sigma Aldrich. Polymeric methylene diphenyl diisocyanate (p-MDI) (≈32 wt% NCO content) was provided by Growchem Sdn. Bhd., Selangor, Malaysia. Silicone surfactant, Tegostab B-8404 was supplied by Evonik Sdn. Bhd. PEG and Gly were mixed with 4:1 volume-to-volume ratio to prepare the PEGGly solvent for liquefaction.

### 2.2. Liquefaction Procedure for Polyol Production

For convenience, the liquefaction method is reproduced in this section, however, the characterizations and optimization of the liquefaction yields and residues have been published elsewhere [19]. In the previous work, liquefaction of EFB fiber was carried out in a refluxed glass reactor with a mechanical stirrer at various temperatures (130, 145, 160, 175, and 190 °C) and times (60, 90, 120, 150, and 180 min). Samples amounting to 150 g of EFB, 750 g of PEGGly solvent, and 22.5 g of H_2_SO_4_ were placed into the reactor. The liquefaction was conducted at the designated temperatures and times with a 600 rpm constant stirring speed. After the reaction, the reactor was instantaneously quenched into cold water. Dioxane-water (4:1 *v*:*v* ratio) solution was used to dilute the liquefied EFB. The mixture was stirred for 30 min before filtered via a polytetrafluoroethylene (PTFE) filter to separate the non-liquefied residues. The filtered liquefied EFB was neutralized using MgO and filtered again for removal of salts. Afterward, the liquefied EFB was heated in a rotary evaporator at 60 °C under reduced pressure to remove the diluent. The remaining liquefied EFB is referred to as EFB polyol. It was found that liquefaction of EFB at 175 °C for 90 min had produced the maximum yield (≈96%) of EFB polyol. Hence, the EFB polyol was used to produce rigid PUFs in this work. The properties of the EFB polyol are listed in the Table 1.

### 2.3. Preparation of PUFs

**Figure 1:**
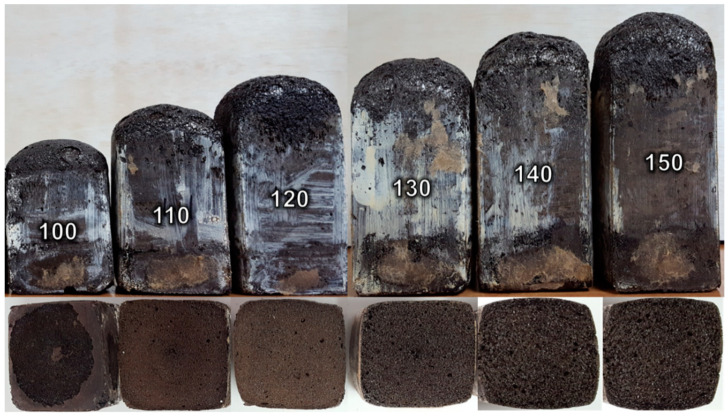
EFBPUFs produced at different NCO indexes.

Rigid PUFs were produced using the EFB polyol via a one-shot foaming method. The PUFs were formulated with different NCO indexes (i.e., 100, 110, 120, 130, 140, and 150). Paper boxes with a dimension of 7 cm × 7 cm × 9 cm (length × width × height) were used as the open molds for the PUFs. Initially, 30 g of the EFB polyol, 0.15 g of DABCO catalyst, 0.75 g of silicon surfactant, and 0.6 g of ultrapure water were pre-mixed and swirled for 2 min. Later, the mixture was added with a preset amount of p-MDI (according to the NCO index), and then stirred at 1000 rpm for a certain time (depending on the cream time of the PUFs at each NCO index—refer to Table 2). The EFB polyol-based PUF produced is denoted as EFBPUF. Figure 1 shows the EFBPUFs produced at different NCO indexes. The quantity of reactants used in the foaming process were determined via Equation (1):

(1) $$\mathrm{isocyanate}\;\mathrm{index}=\left[\frac{M_{p-\mathrm{MDI}}\times m_{p-\mathrm{MDI}}}{\left(M_{\mathrm{Polyol}}\times m_{\mathrm{Polyol}}+\frac{2}{18}\times m_{\mathrm{Water}}\right)}\right]\times 100$$

where M_p−MDI_ = NCO content of p-MDI (converted from 32 wt% of NCO content ≈ 7.62 × 10^−3^ mol/g), m_p−MDI_ = mass of p-MDI (g), M_Polyol_ = OH content of polyol (4.06 × 10^−3^ mol/g), m_Polyol_ = mass of polyol (g), and m_Water_ = mass of water (g).

Calculation example:

$$100=\left[\frac{7.62\times 10^{-3}\times {\;m}_{p-\mathrm{MDI}}}{\left(4.06\times 10^{-3}\times 30+\frac{2}{\;18}\times 0.6\right)}\right]\times 100$$

$$1=\left[\frac{7.62\times 10^{-3}\times {\;m}_{p-\mathrm{MDI}}}{\left(0.19\right)}\right]$$

0.19 = 7.62 × 10^−3^ × m_p–MDI_

m_p__−MDI_ = 24.93 g → mass of p-MDI required for isocyanate index of 100.

### 2.4. Foaming Properties

General foaming properties of the EFBPUFs were evaluated using the ASTM standard for the polyurethane foam cup test [29]. According to the standard, cream time, free-rise time, tack-free time, and free-rise height of the PUFs were measured and determined. The definitions of the terms used in foaming reaction are as below:

(a) Cream time, also known as initiation time, refers to the time between the starting of all reactants being mixed until to the point at which tiny bubbles started to emerge.
(b) Free-rise time refers to the time at when the PUF stops expanding.
(c) Tack-free time is the time at which the surface of the PUF can be touched without sticking.
(d) Free-rise height is the height of PUF at the free-rise time.

### 2.5. Characterization of EFBPUFs

#### 2.5.1. Density of EFBPUFs

The apparent core density of all EFBPUFs was determined in proportion to the Standard Test Method for Apparent Density of Rigid Cellular Plastics—ASTM D1622-14 [30]. The EFBPUF specimens were cut into 6 cm × 6 cm × 6 cm (length × width × height) and measured using a caliper. Then, the specimens were weighed using an analytical balance. The apparent density of the EFBPUFs was computed using Equation (2):

(2) $$D=\frac{m_{s}}{V}$$

where, D = density of specimen (kg/m^3^), m_s_ = mass of specimen (kg), and V = volume of specimen (m^3^).

#### 2.5.2. Morphology of Cells and Cell Size Distribution of EFBPUFs

A scanning electron microscope (SEM-Philips XL-30) was used to observe the morphology of the cellular structure of the EFBPUFs. Five specimens were precisely cut from each EFBPUF, dried, and gold coated before the analysis. The cells sizes of the EFBPUFs were measured from the micrographs in accordance with the procedure proposed by Chen et al. (2015) [31].

#### 2.5.3. Closed-Cell Content

Determination of closed-cell content of the EFBPUFs was carried out using a pycnometer (AccuPyc II 1430, Micromeritics) and nitrogen gas as the displacement medium. This analysis was conducted in accordance with ASTM D6226-15 standard [32]. The following set of equations was used to compute the closed-cell content of the EFBPUFs:

(3) $$O_{v}=\left[\frac{\left(V-V_{\mathrm{Spec}}\right)}{V}\right]\times 100$$

(4) $$W_{v}=\frac{m}{\rho_{\mathrm{Solid}}}\times 100$$

(5) $$C_{v}=100-O_{v}-W_{v}$$

where O_v_ = volume percentage of opened-cell content (%), V = geometrical volume determined using calipers (cm^3^), V_Spec_ = enclosed volume inaccessible by N_2_ gas in pycnometer (cm^3^), W_v_ = volume percentage occupied by cell walls (cell membranes) (%), m = mass of the specimen (g), ρ_Solid_ = density of ground EFBPUF (g/cm^3^), and C_v_ = volume percentage of closed-cell content (%).

#### 2.5.4. Sol Fraction and Degree of Swelling

The sol fraction and degree of swelling of EFBPUFs were determined using the swelling procedure reported by Campanella et al. (2009) [33]. Firstly, EFBPUFs were crushed into coarse powder form and dried in an oven overnight. For the determination of sol fraction, 5 g of each EFBPUF powder was immersed in 200 mL of toluene for 24 h to swell. After that, the swollen EFBPUF powder was filtered and dried. Then, the final weight of the swollen EFBPUF powder was determined. The average results of three specimens from each of the EFBPUFs were recorded. The sol fraction of the EFBPUFs was determined using Equation (6):

(6) $$W_{s}\left(\%\right)=\frac{\left(w_{1}-w_{2}\right)}{w_{1}}\times 100\%$$

where W_s_ = sol fraction of the polymer, w_1_ = weight of the polymer before swelling, and w_2_ = weight of the polymer after swelling.

The volume fraction and degree of swelling of the EFBPUFs were determined via Equations (7) and (8), respectively:

(7) ϕ2=(w2ρ2)[((Was−w2)ρ)+(w2ρ2)]

where, ϕ_2_ = volume fraction of the polymer, W_as_ = weight of the absorbed solvent, w_1_ = weight of the polymer before swelling, w_2_ = weight of the polymer after swelling, ρ = density of solvent, and ρ_2_ = density of the polymer.

(8) $$\mathrm{DS}=\frac{1}{\phi_{2}}$$

where, ϕ_2_ = volume fraction of the polymer.

#### 2.5.5. Fourier Transform Infrared (FTIR) Spectroscopy

FTIR analysis on the EFBPUFs with different NCO indexes was conducted using KBR pellets on a FTIR spectrometer (Bruker, Alpha) with transmittance mode. Each sample was scanned for 64 times at 2 cm^−1^ resolution in the range of 4000–650 cm^−1^. Then, 10 mg of each EFBPUF sample was used for the analysis. The EFBPUF samples were crushed into powder prior to the analysis.

#### 2.5.6. Compression Test

The compressive strength of the EFBPUFs was evaluated using a universal testing machine (Testometric M500-100CT) at a controlled room temperature (20–25 °C) according to ASTM D1621 standard [34]. Specimens were cut from the EFBPUFs into 6 cm × 6 cm × 6 cm (length × width × height) dimensions. The specimens were compressed in a direction parallel to the foam rise. The crosshead speed was set at 2 mm/min using a 10 kN load cell. The compressive strength and modulus of the PUF samples were determined at 10% strain. For comparison, the compressive strength and modulus values of a reference sample of PUF produced using PEG-Gly polyol (100% petro-chemical polyol) at an NCO index of 120 were reproduced from previous publication [19]. The compressive strength of the PUFs was also normalized with their respective densities according to the model for opened- and closed-cell cellular foam used by D’souza et al. (2014) [11]. The normalized compressive strength of the PUFs was calculated using Equation (9):

(9) $$Normalized\;compressive\;strength=\frac{compressive\;strength\;of\;PUF}{density\;of\;PUF^{\frac{3}{2}}}$$

#### 2.5.7. Thermogravimetric Analysis (TGA)

A thermal analyzer (Mettler Toledo TGA/DSC 1 HT 1600) was used to conduct thermogravimetric analysis (TGA) on the EFBPUFs. Then, 10 mg of each EFBPUF sample was heated from ambient temperature to 900 °C with a heating rate of 10 °C/min under N_2_ atmosphere.

## 3. Results and Discussion

### 3.1. Effect of NCO Index on the Foaming Properties of EFBPUFs

The foaming properties of the EFBPUFs produced at different NCO indexes are tabulated in Table 2. The table shows that the cream time of the EFBPUFs decreased when the NCO index was increased. This signifies that the rate of reaction between the EFB polyol and p-MDI increased when a higher NCO index was used. The presence of a higher concentration of p-MDI increased collisions between OHs of polyol with NCO groups in a specific time, hence increasing the rate of reaction. In addition, the reaction of NCO groups with water was more vigorous at higher NCO indexes, generating higher amounts of CO_2_ for the foam to rise. Therefore, the free-rise time of EFBPUFs decreased when higher NCO index was used.

The increase in the rate of reaction also allowed the PU polymers to cure at faster rates. Hence, the tack-free time of the EFBPUFs decreased when the NCO index increased. Principally, the free-rise height of PUFs can be determined by the total amount of the reactants (i.e., polyol and isocyanate). In this regard, the free-rise height of the EFBPUFs increased when the NCO index was increased. However, the free-rise height of the EFBPUFs could also be influenced by the size of cells formed.

Optimization of the NCO index to control foaming reaction is one of the essential steps in developing PUFs. Determination of the optimum NCO index not only produces PUFs with the optimum mechanical properties, but it is also important in selecting favorable foaming and curing reactions. Mild curing and foaming properties are preferable for thermosetting polymeric foams to allow adequate time for proper mixing of the polymer components, as well as to promote well-controlled foaming so that a more isotropic foam can be produced.

### 3.2. Effect of NCO Index on Apparent Density of EFBPUFs

**Figure 2:**
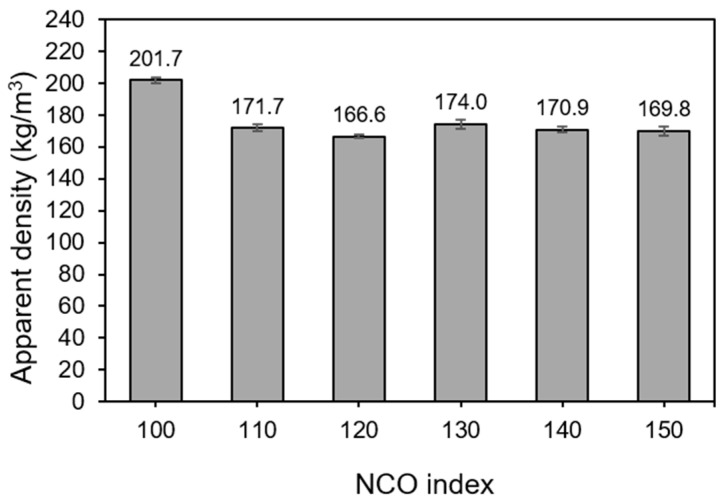
Apparent density of EFBPUFs at different NCO indexes.

Apparent density is one of the important physical properties of PUFs that may affect their mechanical properties. Figure 2 shows the effect of different NCO indexes on the apparent density of EFBPUFs. Based on the results, the apparent density of the EFBPUFs decreased when the NCO index was increased from 100 to 120. There was an increase in apparent density when the NCO index was increased from 120 to 130. However, the apparent density of EFBPUFs decreased again at the NCO index of 130–150.

An increase in the NCO index intensifies the generation of allophanate and biuret groups, as well as the formation of an isocyanurate ring that could increase the crosslink density of PUs [27]. The increase in the crosslink density of PUs might subsequently increase the density of PUFs [35]. However, physical changes (i.e., formation of cells and shrinkages) during the foaming process might have imposed greater impacts on the density of PUFs. Hence, the apparent density of PUFs is not always directly proportional to the NCO index.

The increase in the NCO index also increased the rate of the foaming reaction of the EFBPUFs. During foaming, NCOs reacted with the OH groups of EFB polyol to form urethane and allophanate linkages. Besides, NCOs also reacted with water (blowing agent) to produce amines, ureas, and biurets (secondary reaction between NCOs and ureas), with CO_2_ and heat generated as by-products. In addition, dimerization and trimerization of NCOs would produce carbodiimide and isocyanurate groups, respectively. Thus, the rates of crosslinking (gelation) and CO_2_ evolution (blowing) would increase at higher rates than the NCO index. If gelation is faster than the blowing reaction, closed-cells will be produced. Meanwhile, if the blowing reaction is faster than the gelation of PUF, opened-cells will be formed [36].

In a cellular polymeric structure such as PUF, the morphological analysis could further explain its apparent density. PUFs with larger cell size or/and a greater number of opened cells would have lower apparent density than the PUFs with smaller cell size or/and a lesser number of opened cells. The decrease in apparent density of EFBPUFs from the NCO index of 100 to 120 might have been due to the enlargement of cells and formation of more opened cells. However, information on the cellular structure morphology of the EFBPUFs is essential to understand the increase in apparent density from the NCO index of 120 to 130.

### 3.3. Cellular Morphology and Cell Size Distribution of EFBPUFs

**Figure 3:**
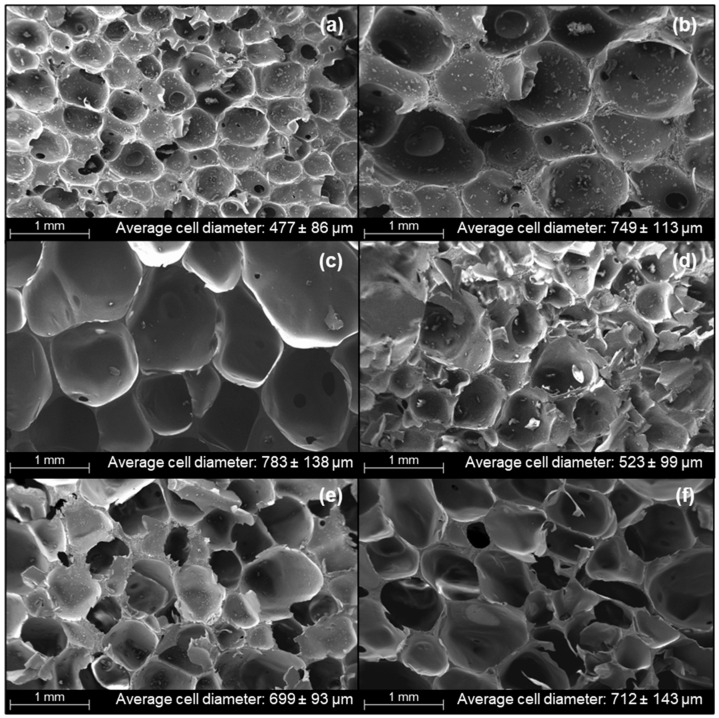
SEM micrographs of EFBPUFs synthesized using different NCO indexes (**a**) 100, (**b**) 110, (**c**) 120, (**d**) 130, (**e**) 140, and (**f**) 150.

Cellular morphology and cell sizes are important physical properties that could influence the mechanical properties of PUFs. In this analysis, the cell size of EFBPUFs is presented in the form of cell diameter. The effect of different NCO indexes on the cellular morphology of EFBPUFs is shown in Figure 3. Based on the micrographs, the cell size of EFBPUF increased when the NCO index was increased from 100 to 120 (Figure 3a–c). This explains the decrease in the apparent density of the EFBPUFs, as discussed in Section 3.2. The average cell diameter increased substantially with the increase of the NCO index from 100 to 110 (477.21 to 749 µm) (Figure 3a,b). The increase in the NCO index had increased the curing rate and the amount of CO_2_ released during foaming, thus, the cellular size became larger. Nonetheless, almost all the cells in the EFBPUFs at the NCO index of 100 and 110 were closed cells, indicating that the diffusion of gases through curing PU networks occurred at a steady rate.

As the NCO index was increased from 110 to 120, the cells became slightly larger, with the average cell diameter increasing from 749 to 783 µm (Figure 3b,c). This slight increase in cell size might have also been caused by the increase in curing rate and amount of CO_2_ released. However, more opened cells were present in the EFBPUF at the NCO index of 120 compared to that of 110. This signifies that at the NCO index of 120, the release of gases was faster than the crosslinking of PU networks [36]. The cell structures, and cell sizes of EFBPUFs at these NCO indexes (110 and 120), indicate that the optimum NCO index to produce EFBPUFs might lie within this range.

**Figure 4:**
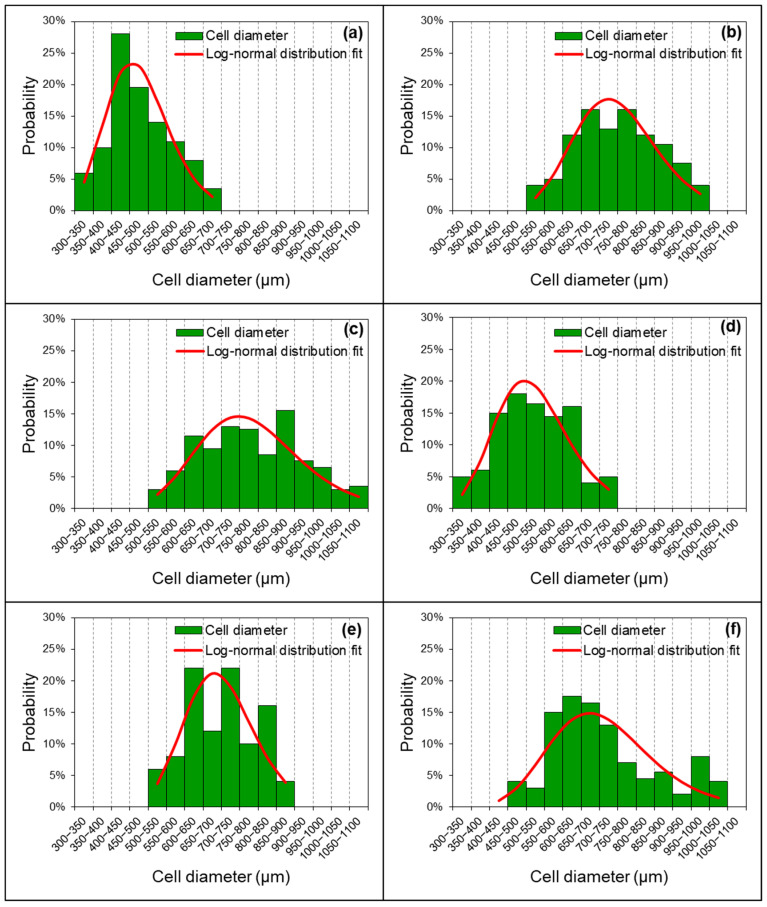
Cell size distribution of the EFBPUFs at different NCO indexes (**a**) 100, (**b**) 110, (**c**) 120, (**d**) 130, (**e**) 140, and (**f**) 150.

When the NCO index was increased from 120 to 130 (Figure 3c,d), the average cell diameter of EFBPUF decreased remarkably (from 783 to 523 µm). At the NCO index of 130, the cell size became smaller and most of the cells were closed cells (Figure 3d). The cells were also filled with solid polymer. At this stage, the increase in the NCO index had further increased the curing rate of EFBPUF, but the higher rate of gases released had simultaneously prevented the development of opened cells [36]. Excessed p-MDI trapped within the cells filled the cellular cavity and hardened into solid polymer, causing the cell size reduction. As the NCO index increased from 130–150 (Figure 3d–f), the average cell diameter increased from 523 to 712 µm. The average cell diameters for the EFBPUFs at the NCO indexes of 140 and 150 were 699 and 711 µm, respectively. Most of the cells in both EFBPUFs produced at the NCO indexes of 140 and 150 showed opened-cell structures (Figure 3e,f), and this was one of the reasons for the decrease in compressive strength of the EFBPUFs. Figure 4 shows the distribution of cell diameters of EFBPUFs produced at different NCO indexes. The results showed that the measured cell diameters of all EFBPUFs followed a log-normal distribution. A larger distribution of cell diameters signifies that the PUF possessed a greater range of cell diameters.

### 3.4. Sol Fraction and Degree of Swelling of EFBPUFs

**Figure 5:**
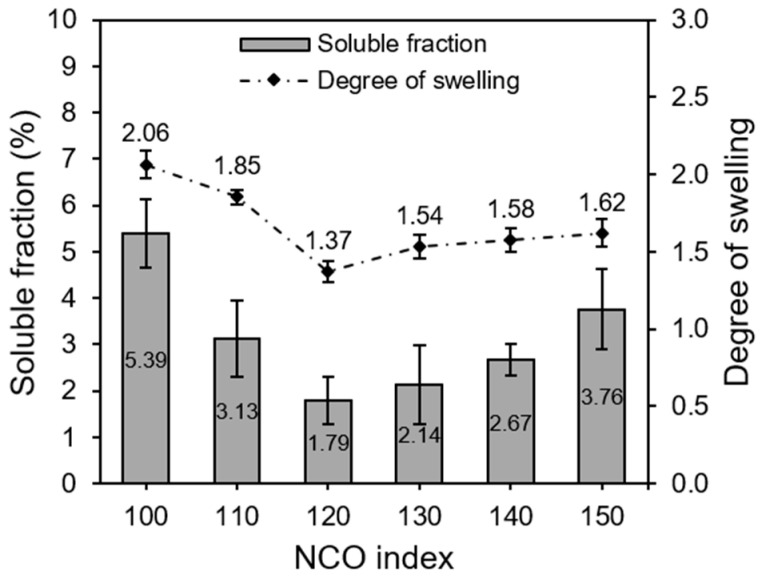
Sol fraction (%) and degree of swelling of the EFBPUFs at different NCO indexes.

Crosslink formation in a thermoset polymer is essential in determining its key properties. Higher crosslink density imparts higher rigidity for a polymer and vice versa. The crosslink density of a PU could be indirectly assessed via a useful metric, namely the degree of swelling. When a crosslinked polymer absorbs solvent, the polymer networks restrict its expansion. Thus, a lower degree of swelling signifies higher crosslink density. Another approach used to analyze the crosslinking of EFBPUFs is by determining the sol fraction (%). During solvent treatment, polymer chains that were not crosslinked would leach out into the solvent. Hence, lower sol fraction indicates higher crosslink density. Figure 5 shows the sol fraction (%) and degree of swelling of the EFBPUFs produced at different NCO indexes.

The results showed that the sol fraction and swelling degree of EFBPUFs decreased when NCO index was increased from 100 to 120. Then, the sol fraction and swelling degree of EFBPUFs increased with the NCO index from 120 to 150. The EFBPUF produced at the NCO index of 100 showed the highest sol fraction and swelling degree. This might be due to an insufficient amount of NCOs present to crosslink all OH groups in EFB polyol. Although the molar ratio of NCO/OH was equal to 1.0 at the NCO index of 100, some of the NCOs might have been lost during the foaming process and not reacted with EFB polyol to produce PU networks. Hence, high amounts of unreacted EFB polyol chains leached out into the solvent. The increase in crosslink density might have decreased the sol fraction and swelling degree of EFBPUFs (NCO index = 100–120). Side reactions that occurred in the presence of excessed NCOs could have increased crosslink density of PUs via formation of multifunctional (i.e., bifunctional and trifunctional) groups, such as urea, biuret, allophanate, and isocyanurate [37]. Besides, the increase in the NCO index had also increased the amount of hard segments in the PU network, limiting solvent penetration between the PU chains.

Further increases in the NCO index (from 120 to 150) increased the sol fraction and swelling degree of EFBPUFs. The incremental increase of the sol fraction and swelling degree might be due to the dissolution of higher amounts of excessed p-MDI as the NCO index increased. There are two gel points for a diol-diisocyanate system, where low levels of excessed NCOs promote sol–gel transition and whereby high levels of excessed NCOs result in gel–sol transition. Based on our results, the transition between the two gel points showed at the NCO index of 120.

### 3.5. FTIR Analysis

**Figure 6:**
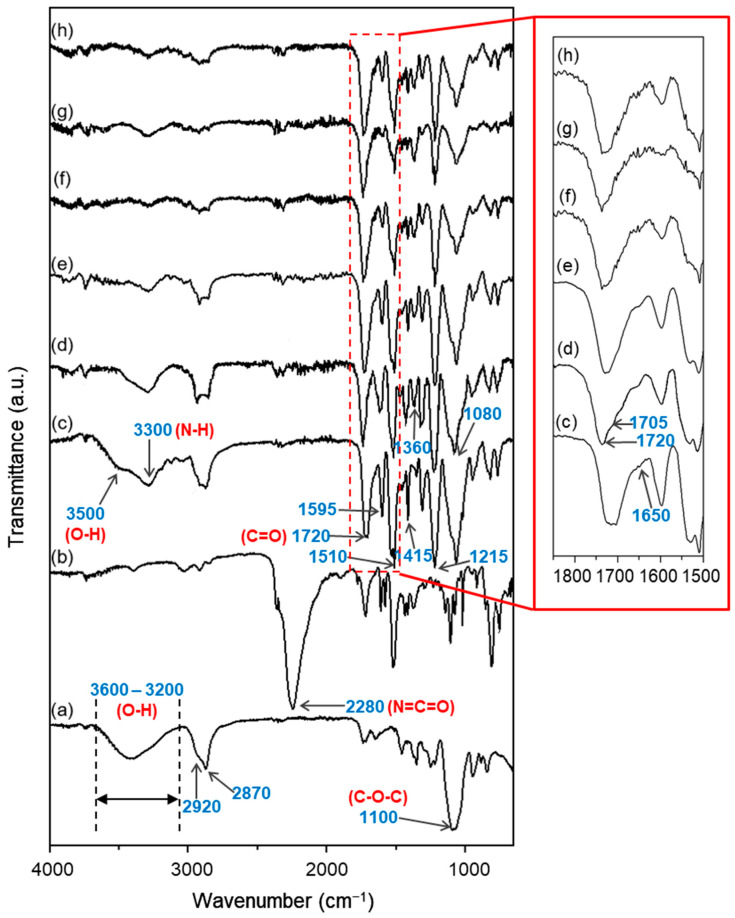
FTIR spectra of (**a**) EFB polyol, (**b**) p-MDI, and EFBPUFs at different NCO indexes of (**c**) 100, (**d**) 110, (**e**) 120, (**f**) 130, (**g**) 140, and (**h**) 150.

The effect of different NCO indexes on the chemical structure of the EFBPUFs was studied by analyzing their functional groups using FTIR analysis. The infrared (IR) spectra of the EFB polyol, p-MDI, and EFBPUFs produced at different NCO indexes are shown in Figure 6. The main reaction in the production of a PU is the reaction between OH groups of a polyol with the NCO groups of the diisocyanates (p-MDI) to form urethane linkages. Besides urethane as the repeating groups, a PU also contains urea, ether, ester, and aromatic compounds in its structure [37].

In Figure 6a, a broad peak at 3600–3200 cm^−1^ (stretching vibration of O–H) and peaks at 2920 cm^−1^ (asymmetric stretching vibration of C–H), 2870 cm^−1^ (symmetric stretching vibration of C–H), and 1100 cm^−1^ (C–O–C) belong to the EFB polyol. Common peaks that represent functionalities of urethanes and ureas in PU, such as a broad peak at 3300 cm^−1^ (stretching vibration of amine (N–H) in urethane linkages); peaks at 1720 cm^−1^ (free C=O of urethanes), 1705 cm^−1^ (H-bonded C=O of urethanes), 1595 cm^−1^ (aromatic group of p-MDI), 1510 cm^−1^ (bending vibration of N–H), 1415 cm^−1^ (isocyanurate ring), 1360 cm^−1^ (aromatic amine C−N stretching from p-MDI) [38], 1215 cm^−1^ (stretching vibration of C–N in urethane linkages) [27], and 1080 cm^−1^ (C–O–C of urethanes); and a shoulder peak at 1650 cm^−1^ (N–H of urea) [39] are present in all EFBPUFs spectra.

The peak at 3500 cm^−1^ (O–H) present in the spectrum of EFBPUF at the NCO index of 100 (Figure 6c) signifies the presence of unreacted OH groups from the EFB polyol. Although the molarity of NCO/OH used at this NCO index was equivalent to ≈1.0, some amount of NCOs might have been consumed via reaction with water (blowing agent) during the foaming reaction, causing the remaining NCOs left to be inadequate at completely crosslinking the OH groups. Peaks at 3600–3200 cm^−1^ (O–H groups) from the EFB polyol (Figure 6a) and 2280 cm^−1^ (stretching vibration of N=C=O from the p-MDI) (Figure 6b) were diminished and disappeared in the spectra of EFBPUFs at the NCO indexes of 110–150 (Figure 6d–h). There was no peak present at 2280 cm^−1^, implying that no free NCOs were detected in all of the EFBPUF samples. The removal of free and unreacted NCOs might have occurred during the post-cure heating (at 120 °C for 1 h) of the EFBPUFs.

For the fully crosslinked EFBPUFs (NCO index 110–150), the intensity of an ether (C–O–C of urethanes) peak at 1080 cm^−1^ represents the extent of urethane linkages formed. There was a slight increase in the peak (1080 cm^−1^) intensity when the NCO index was increased from 110 to 120 (Figure 6d,e). Meanwhile, the EFBPUFs at the NCO index of 130 to 150 (Figure 6f–h) showed lower intensity of the peak than the EFBPUF at the NCO index of 120. It is known that at the NCO indexes higher than 100, the NCOs present were sufficient to completely crosslink all the OHs in the EFB polyol to form urethane linkages. Thus, the formation of the urethanes might have achieved the saturation point within the NCO indexes of 110 and 120.

The free carbonyl (C=O) peak (1720 cm^−1^) of urethanes shown in the EFBPUFs spectrum represents the interaction between hard segments (urethanes) and soft segments (ethers or esters of the polyol), whereas the H-bonded C=O peak (1705 cm^−1^) represents the intermolecular hydrogen bonding between hard segments (inter-urethane H-bonding) [6]. From the result, the peak intensity of 1705 cm^−1^ increased as the NCO index was increased from 110 to 120 (Figure 6d,e), forming a blunt peak (1720 and 1705 cm^−1^), implying that more H-bonded C=O was formed. The peak intensity remained relatively unchanged as the NCO index was increased from 120 to 150 (Figure 6e–h). This analysis suggests that the EFBPUF at the NCO index of 120 had the highest intermolecular interaction between its hard segments.

### 3.6. Compressive Properties of EFBPUFs

Compressive properties of a cellular polymer result from complex mechanisms due to the presence of voids and an interconnected network of solid plates or struts, which form cell walls (cell membranes) and edges. Principally, mechanical properties of most PUFs are closely correlated to their densities and types of cells (closed and/or opened cells).

Linear elastic deformation of struts in a rigid PUF to withstand the applied compressive force is the main factor that determines the compressive modulus and strength of the PUF. However, pressurization of enclosed gas and stretching of the cell membrane are the additional mechanisms that occur in a closed-cell PUF. In estimation, the enclosed gas would provide maximum pressurization of 101 kPa (≈1 bar) if the PUF was produced under atmospheric pressure. At 10% compressive strain, it is estimated that only ≈10 kPa (≈0.01 MPa) of enclosed gas pressurization would be produced from the total compressive modulus of the closed-cell PUF. However, when compared to the high compressive modulus of the EFBPUFs, the effect of gas pressurization was insignificant in evaluating the compressive strength.

**Figure 7:**
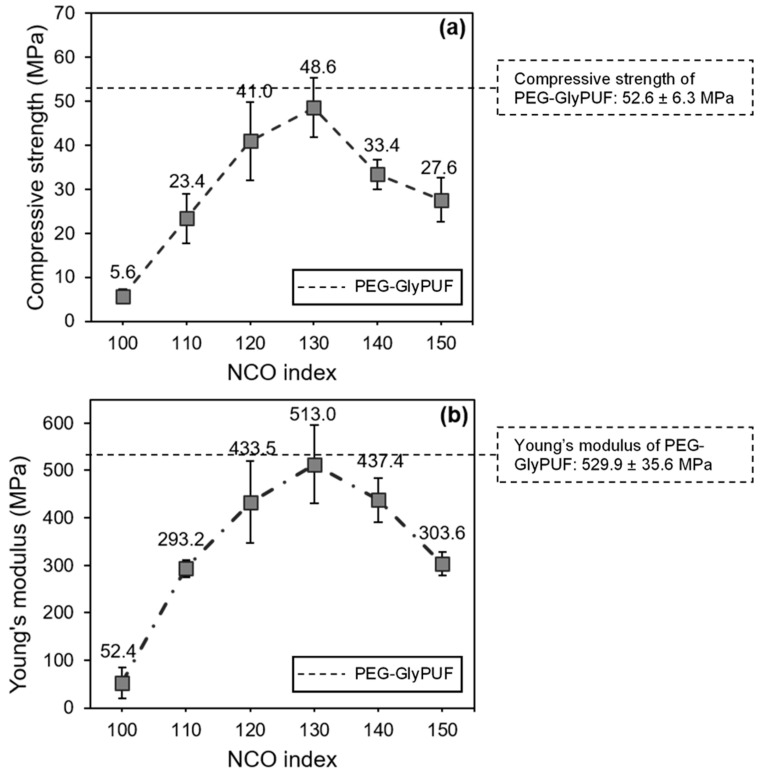
Compressive (**a**) strength and (**b**) modulus of EFBPUFs at different NCO indexes with PEG-GlyPUF as the reference sample.

The effects of different NCO indexes on the compressive strength and Young’s modulus of EFBPUFs are shown in Figure 7a,b, respectively. Additional data on apparent density, closed-cell content, compressive modulus, and normalized compressive strength of the EFBPUFs are presented in Table 3. The values for physical and compressive properties of PEG-GlyPUF (100% PEG-Gly polyol-based PUF produced at NCO index of 120) obtained from a previous publication [19] are also presented in the results as the reference. Based on the results shown in Figure 7a, the compressive strength of the EFBPUFs increased at the NCO indexes of 100–130. However, the compressive strength decreased when NCO index was increased from 130 to 150. In Figure 7b, the compressive Young’s modulus of EFBPUFs showed a similar trend with the compressive strength. The increase in compressive strength (NCO index = 100–130) might be due to the increase in crosslinking of the PU networks. Higher crosslinking PU networks would produce PUFs with higher rigidity. Therefore, the compressive strength of the EFBPUFs would increase. Crosslinking of PU networks in EFBPUFs at the NCO indexes of 130–150 might also increase, but their compressive strength would decrease. The decrease in the compressive strength of EFBPUFs (NCO index = 130–150) seems to be more relatable with their foaming properties. As discussed in Section 3.1, the rate of foaming of EFBPUFs increased when NCO index was increased. However, the rate of foaming at the NCO index of 130–150 might be extremely high, producing large cells and more opened cells, which decreased the compressive strength and modulus. The compressive strengths of the EFBPUFs were also normalized with their respective densities to provide a more accurate and equitable comparison. The normalized compressive strengths of the EFBPUFs also showed similar trends with the original compressive strength. However, it was shown that the difference in normalized compressive strengths of EFBPUFs at the NCO indexes of 120 (19.0 kPa/(kg/m^3^)^3/2^) and 130 (21.2 kPa/(kg/m^3^)^3/2^) was exceedingly small. Comparatively, the results showed that the EFBPUFs at all NCO indexes had lower compressive strength and modulus than that of the reference PEG-GlyPUF (NCO index = 120). The OH content of the EFB polyol (4.06 × 10^−3^ g/mol) was lower than the value reported for the PEG-Gly polyol (6.6 × 10^−3^ g/mol) [19]. Hence, the EFBPUFs might have had lower crosslinking density than the PEG-GlyPUF, which had contributed to the decrease in the compressive strength and modulus. Nonetheless, at the NCO index of 120, the normalized compressive strength of the EFBPUF (19.0 kPa/(kg/m^3^)^3/2^) was just slightly lower compared to that of PEG-GlyPUF (21.7 kPa/(kg/m^3^)^3/2^).

### 3.7. Thermogravimetric Analysis of EFBPUFs

**Figure 8:**
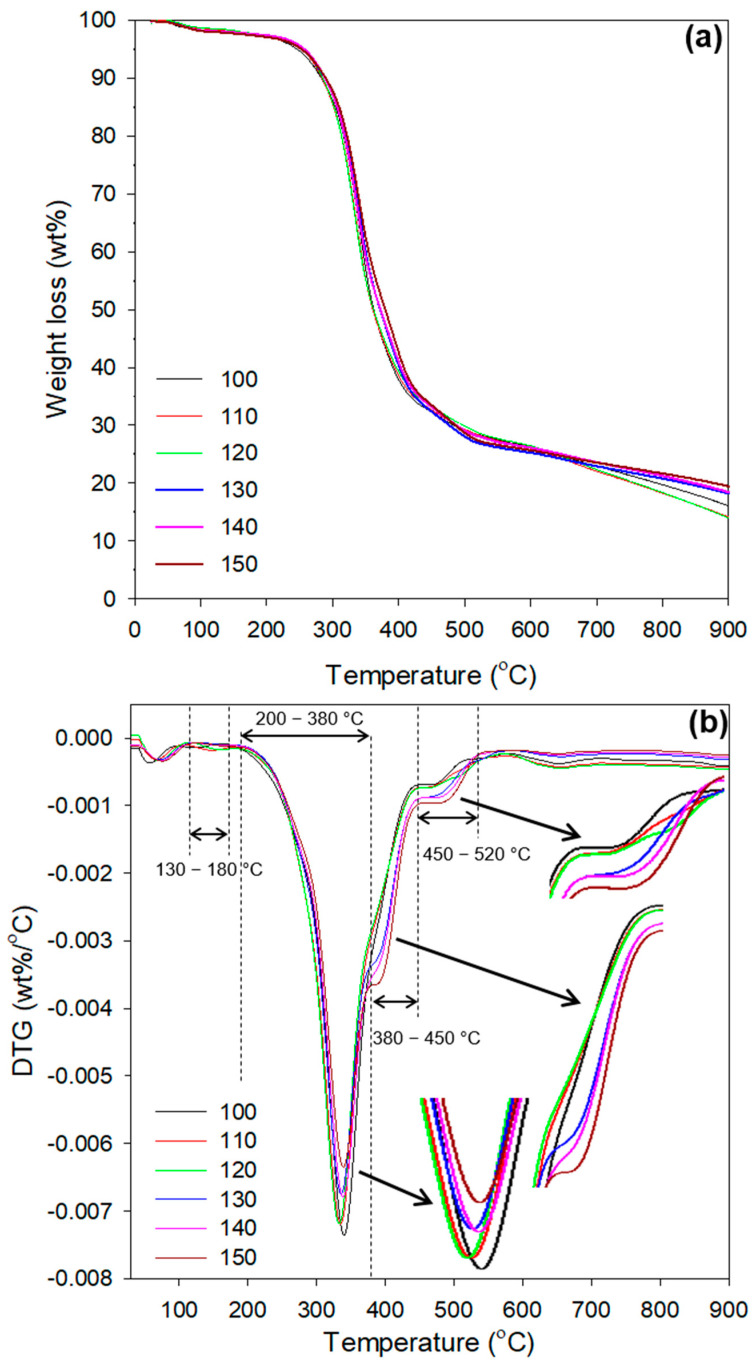
(**a**) TGA and (**b**) DTG curves of EFBPUFs produced at different NCO indexes.

Polyurethane (PU) undergoes a complex thermal degradation, which includes several partial degradation reactions. The mechanism of thermal degradation of PU involves random-chain and chain-end scissions, as well as crosslinking. A bulky crosslinked PU requires high heat energy to break its bonds. Mainly, thermal degradation of PUs occurs in two (in N_2_ environment) or three (in oxidizing environment) stages [40]. At first, urethane linkages or hard segments are degraded. Afterwards, the degradation of polyols or soft segments (ether and ester linkages) occurs at the second and third stages [37]. Figure 8a,b portray the thermogravimetric (TGA) and derivative thermogravimetric (DTG) curves of EFBPUF produced at different NCO indexes, respectively.

The TGA curves for all EFBPUFs showed that only one stage of degradation occurred (Figure 8a). From the figure, the degradation started at around 60 °C and extended up to around 100 °C. The largest weight loss commenced at 200 °C, showed by the steep declining curves. The curves begin to shallow at 550 °C, but small and gradual weight loss continued up to 900 °C. According to the TGA curves, the two stages of degradation cannot be clearly seen. However, the DTG curves present distinct peaks that could provide more information regarding the thermal degradation of the EFBPUFs (Figure 8b).

In Figure 8b, the first stage of degradation is shown by a small peak at 60–100 °C in all EFBPUFs. This peak represents the release of volatile materials such as gases and water. Another small peak found at 130–180 °C is attributed to phenolic urethanes, which are degraded at a lower temperatures than aliphatic urethanes [41]. The adjacent large peak at 200–380 °C constitutes the degradation of urethane, urea, and isocyanurate linkages (hard segments), which occurred at 200, 250, and 350 °C, respectively. The degradation of these linkages resulted in the formation of polyols (soft segments) and free NCOs, primary or secondary amine and olefin, and CO_2_ [15,37]. After the degradation of the main PU linkages, i.e., urethanes at ca. 200 °C, the NCOs that were formed underwent dimerization and trimerization into carbodiimides and isocyanurates, respectively. These products (carbodiimides and isocyanurates) of the side reactions (dimerization and trimerization) might have crosslinked with the released polyols to produce substituted ureas and polyisocyanurates, which are relatively more stable.

The EFBPUFs showed different peak intensities in this range of temperature (200–350 °C). Intensities of the peak with respect to the NCO indexes of the EFBPUFs, in ascending order, were, 150 (brown) < 130 (blue) < 140 (pink) < 120 (green) < 110 (red) < 100 (black). However, the intensities of the peaks for the NCO indexes of 110, 120, 130, and 140 were very close to each other. The order represents an increasing amount of weight loss due to the evaporation of NCOs after the degradation of urethane linkages in the EFBPUFs produced at the respective NCO indexes. It also indicates that a greater amount of urethane linkages were degraded at lower NCO indexes. Thus, it can be said that the thermal stability of the EFBPUF increased when the NCO index was increased.

Jiao et al. (2013) used TGA-FTIR-mass spectroscopy (TGA-FTIR-MS) to analyze volatile substances from thermal degradation of PUs at various temperatures [40]. They found that when the temperature was further increased, the polyols formed via the degradation of urethane linkages degraded into aliphatic ether alcohols. FTIR results of evolved gaseous products showed functional groups of degradation products for polyether segment of polyols such as CH, CH_2_, CH_3_, and C–O–C stretching vibrations at 250–360 °C. At 320–570 °C, OH compounds, carbonyl (C=O) groups of esters, and amine (N–H) groups of aromatic secondary amines were also identified. The evolution of CO_2_ was detected in two stages, namely at 250–410 °C (degradation of urethanes) and 410–670 °C (degradation of polyols). Other products, such as extremely toxic and poisonous hydrogen cyanide (HCN) and nitrogen dioxide (NO_2_), were also found via mass spectrometer. HCN was detected at around 365 °C [40].

In this study, the peak showed at 380–450 °C might represent the evaporation of HCN. Meanwhile, the first stage of evolution of CO_2_ might have overlapped with the release of NCOs at 200 °C and extended until 450 °C (200–450 °C), causing the formation of a shoulder peak at 380–450 °C. Afterwards, the second evolution of CO_2_ is represented by the peak at 450–520 °C (Figure 8b). Different intensities of the shoulder peak showed at 380–450 °C represent the release of different amounts of CO_2_ and HCN by EFBPUFs at different NCO indexes. The intensity of the peak decreased from the NCO index of 100–120. This indicates that a lesser amount of HCN had been released when the NCO index was increased from 100–120. The decrease in HCN released might be due to the increase in the crosslink density of the EFBPUFs. However, when the NCO index was increased from 120 to 150, the intensity of the shoulder peak increased, indicating a higher amount of HCN had been released. The increase in the NCO index increased the amount of nitrogen-containing compounds in the EFBPUFs, hence, resulting in the formation of more HCN.

The second shoulder peak, showed at 450–520 °C, is attributed to the second stage of CO_2_ evolution. The intensity of the peak increased with the NCO index, signifying that a greater amount of the initially released polyols were re-crosslinked into more thermally stable compounds at higher NCO indexes. Hence, a higher portion of polyols degraded at this range of temperature (450–520 °C), releasing a greater amount of CO_2_ in the second stage. When the temperature was further increased to 900 °C, char was produced. From the DTG curves, EFBPUF produced at the NCO index of 120 showed the highest amount of char. This indicates that the EFBPUF (NCO index of 120) had the highest amount of carbon derived from its combination of raw materials. However, the EFBPUFs produced at the NCO indexes of 130–150 showed a decrement of char content. It signifies that the EFBPUFs contained a higher amount of nitrogen compounds that evaporated as the NCO index increased from 130–150.

## 4. Conclusions

Rigid EFBPUFs were produced at different NCO indexes using liquefied EFB bio-based polyol. The effect of different NCO indexes on the foaming, physical, and chemical properties of the EFBPUFs was studied. Each EFBPUF showed a unique set of properties at different NCO indexes. Foaming properties affected the density and cellular morphology of the EFBPUFs, which later influenced their compressive properties. The EFBPUF produced at the NCO index of 100 (molar ratio NCO/OH = 1.0) was not fully crosslinked. The highest normalized compressive strength of the EFBPUFs could be obtained at the NCO indexes of 120–130, since the difference was relatively very small. The normalized compressive strength of the EFBPUF at the NCO index of 120 was also comparable with the strength of PUF produced using petrochemical-based polyol (PEGGlyPUF). The thermal stability of EFBPUFs increased as the NCO index was increased. The smallest emission of highly toxic and poisonous HCN by the EFBPUF was at the NCO index of 120. It is proposed that EFBPUFs with optimum physical, compressive, and chemical properties can be produced at the NCO index of 120. This study allows cleaner production of PUFs by reducing petroleum-based polyol dependency with minimum consumption of isocyanate, while simultaneously harnessing the potential of biomass-based chemicals via utilization of oil palm plantation byproducts.

## Figures and Tables

**Table 1:** Properties of the EFB polyol.

Polyol	OH Content (mol/g)	M_w_ (Da)	Viscosity (cP)
EFB polyol	4.06 × 10^−3^	698	858

**Table 2:** Foaming properties of EFBPUFs at different NCO indexes.

NCO Index	Cream Time (s)	Free-Rise Time (s)	Tack-Free Time (s)	Free-Rise Height (cm)
100	21.4	92.5	123.2	9.1
110	13.8	88.1	110.3	11.1
120	9.2	84.8	102.0	12.3
130	7.4	72.3	86.8	13.5
140	6.1	64.1	76.6	14.6
150	5.5	52.4	63.4	15.2

**Table 3:** Physical and compressive properties of EFBPUFs at different NCO indexes with PEG-GlyPUF as the reference sample.

NCO Index	Closed-Cell Content (%)	Density (kg/m^3^)	Compressive Modulus at 10% Strain (MPa)	Normalized Compressive Strength [kPa/(kg/m^3^)^3/2^]
PEG-GlyPUF	67.7 ± 1.3	180.7 ± 1.7	518.3 ± 55.3	21.7 ± 0.3
100	70.7 ± 3.4	201.7 ± 2.1	51.1 ± 13.4	1.9 ± 0.1
110	64.0 ± 1.3	171.7 ± 1.3	250.3 ± 66.9	10.4 ± 0.2
120	58.6 ± 2.7	166.6 ± 3.0	410.8 ± 95.7	19.0 ± 0.5
130	66.6 ± 4.0	174.0 ± 1.6	506.4 ± 105.7	21.2 ± 0.3
140	41.3 ± 3.8	170.9 ± 3.1	380.5 ± 103.5	14.9 ± 0.4
150	39.0 ± 1.6	169.8 ± 3.1	284.1 ± 50.0	12.5 ± 0.2
